# A Genome-Wide Comparative Evolutionary Analysis of Herpes Simplex Virus Type 1 and Varicella Zoster Virus

**DOI:** 10.1371/journal.pone.0022527

**Published:** 2011-07-25

**Authors:** Peter Norberg, Shaun Tyler, Alberto Severini, Rich Whitley, Jan-Åke Liljeqvist, Tomas Bergström

**Affiliations:** 1 Department of Virology, University of Gothenburg, Gothenburg, Sweden; 2 National Microbiology Laboratory, Public Health Agency of Canada, Canadian Science Centre for Human and Animal Health, Winnipeg, Canada; 3 University of Alabama at Birmingham, Birmingham, Alabama, United States of America; Institute of Infectious Disease and Molecular Medicine, South Africa

## Abstract

Herpes simplex virus type 1 (HSV-1) and varicella zoster virus (VZV) are closely related viruses causing lifelong infections. They are typically associated with mucocutaneous or skin lesions, but may also cause severe neurological or ophthalmic diseases, possibly due to viral- and/or host-genetic factors. Although these viruses are well characterized, genome-wide evolutionary studies have hitherto only been presented for VZV. Here, we present a genome-wide study on HSV-1. We also compared the evolutionary characteristics of HSV-1 with those for VZV. We demonstrate that, in contrast to VZV for which only a few ancient recombination events have been suggested, all HSV-1 genomes contain mosaic patterns of segments with different evolutionary origins. Thus, recombination seems to occur extremely frequent for HSV-1. We conclude by proposing a timescale for HSV-1 evolution, and by discussing putative underlying mechanisms for why these otherwise biologically similar viruses have such striking evolutionary differences.

## Introduction

Human alpha-herpesviruses comprise three members, herpes simplex virus (HSV) 1 and 2 and varicella zoster virus (VZV). These viruses have a wide host cell range and an efficient and rapid reproductive cell cycle [1]. HSV-1 is typically associated with oral lesions, but primary genital HSV-1 infections have recently become more commonly detected [2]. HSV-2 is the one of the most common sexually transmitted pathogens and is typically associated with genital lesions, although primary oral HSV-2 infections may occur. VZV is the causative agent of chickenpox. After infection, all three viruses have the capacity to establish life-long infections with latency/persistency in sensory neurons, and may induce vesicular lesions during reactivation; HSV-1 and HSV-2 in form of oral or genital ulcers while VZV cause herpes zoster. In addition, these viruses may cause severe complications from the visual and nervous systems. Genetic markers of virulence among circulating strains are largely unknown, but are suggested to be involved in the pathogenesis [3], [4], [5].

The genomes of HSV and VZV contain a unique long (UL) and a unique short (US) region, each flanked with inverted repeat regions [6], [7]. Previous analysis of the three genes US4, US7 and US8, located in the US region and coding for the glycoproteins G (gG), I (gI) and E (gE), respectively, demonstrated a division of HSV-1 strains into three clades, designated as A, B and C [8]. Furthemore, 22% of the strains were classified as recombinants. An RFLP genotyping assay was also developed to classify HSV-1 strains to any of the three clades [9]. This assay has subsequently been utilized in e.g. genotype-phenotype association studies [10], and analysis of geographic stability of HSV-1 genotypes [11]. Although recombination crossovers have been demonstrated for sub-genomic regions [8], [12], the extent of recombination in HSV-1 considering complete genomes has hitherto been unknown.

Studies of whole genome HSV-1 sequences are rare, and only three complete HSV-1 genomes are currently available in GenBank. These are strain 17, which was sequenced over 20 years ago [13], [14], and strains F and 129 which were sequenced in 2010 [15]. Similarly, only one HSV-2 strain, HG52 (GenBank accession # NC_001798), is currently available in GenBank. Consequently, no genome-wide phylogenetic analysis based on HSV-1 or HSV-2 has hitherto been presented. In contrast, the complete genome sequence of VZV is well established, and analysis of VZV has demonstrated that globally circulating strains cluster to distinct clades in phylogenetic trees [16], [17], [18]. Although homologous recombination has contributed to the evolution of VZV, these events were rare and seemed to be relatively old since all members in a specific phylogenetic clade shared the same patterns of recombination crossovers. Therefore, it is possible to genotype a complete VZV genome by analyzing one or a few loci and several such studies have been reported [16], [19]. Here, as part of a search for possible genetic traits of neurovirulence in HSV-1, we sequenced nine HSV-1 genomes. In total, ten clinical and two laboratory HSV-1 strains were included in the analysis. We found a striking difference to VZV genomics in that each HSV-1 strain appeared as mosaic recombinants. We propose a time-scale for HSV-1 evolution that demonstrates a slow divergence into different clades starting ∼700.000 years before present (BP). The findings allowed a comparison of the genetic evolution of these two related human alpha-herpesviruses.

## Results

### Genetic diversity

**Figure 1:**
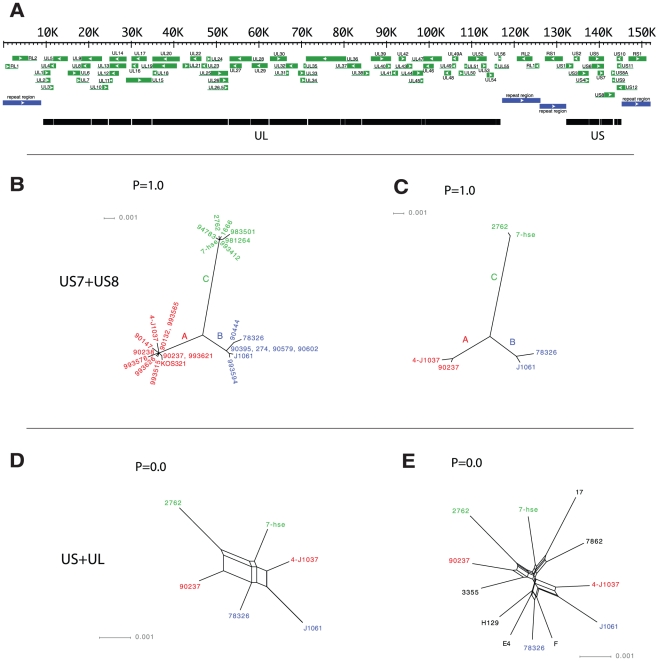
HSV-1 phylogeny. (A) Schematic illustration of the HSV-1 genome. The thick black bar in the bottom depicts the regions that were included in the analysis after all repeat regions and gaps were removed. Nucleotide positions refer to the laboratory strain 17. (B, C, D and E) Phylogenetic networks (splits networks). Networks B and C are based only on US7 and US8, which were the genes analyzed previously by Norberg et al [8]. All strains with recombination crossovers in this region were excluded in order to illustrate the division of the non-recombinant strains into the three distinct evolutionary clades A, B and C. Network B also includes the strains used by Norberg et al, and network C includes only the strains sequenced for this study. Network D is based on the complete genome, including only the strains used in network C (i.e. without recombination crossovers in US7 or US8). Here, recombination crossovers in other parts of the genome prohibit distinct classification of the strains into three distinct clades. Finally, network E is based on the complete genome, including all strains. The network reveals massive recombination and it is irrelevant to attempt to classify a complete genome into any of the three phylogenetic clades A, B or C. The statistical significance for recombination is shown for each dataset.

A total of 119,727 nucleotides were analyzed in each HSV-1 genome. A schematic illustration of the genome regions analyzed is given in Fig. 1A. The genetic distance between globally circulating strains was estimated for HSV-1 and VZV. The genetic distance between the most distantly related HSV-1 strains ranged from approximately 0.4% to 1.7%, and the most distantly related VZV strains ranged from approximately 0.1% to 0.5%. The US region was more variable than the UL region for both viruses.

### Phylogenetic and recombination analysis

Two splits networks were initially constructed based on the US7 to US8 region. The first network (Fig. 1B) includes all strains without recombination crossovers in the US7 to US8 region previously analyzed by Norberg et al [8]. The second network (Fig. 1C) includes only strains which were sequenced in this study, and where no recombination crossovers were detected in the US7–US8 region. These two networks clearly divide the strains into the three previously described clades A, B and C with no statistical significance for recombination (p = 1.0). Two genome-wide networks were subsequently constructed. The first (Fig. 1D) only includes strains without recombination crossovers in the US7–US8 region. There was, however, a high statistical significance for recombination in the dataset (p = 0.0), and the recombination crossovers prohibited a distinct classification of the strains into clades A, B and C. The genome-wide network based on all strains (Fig. 1E) presented frequent recombination crossovers in the genomes that entirely erased the classification into distinct clades, leaving a star-shaped phylogeny and a high statistical significance for recombination (p = 0.0).

**Figure 2:**
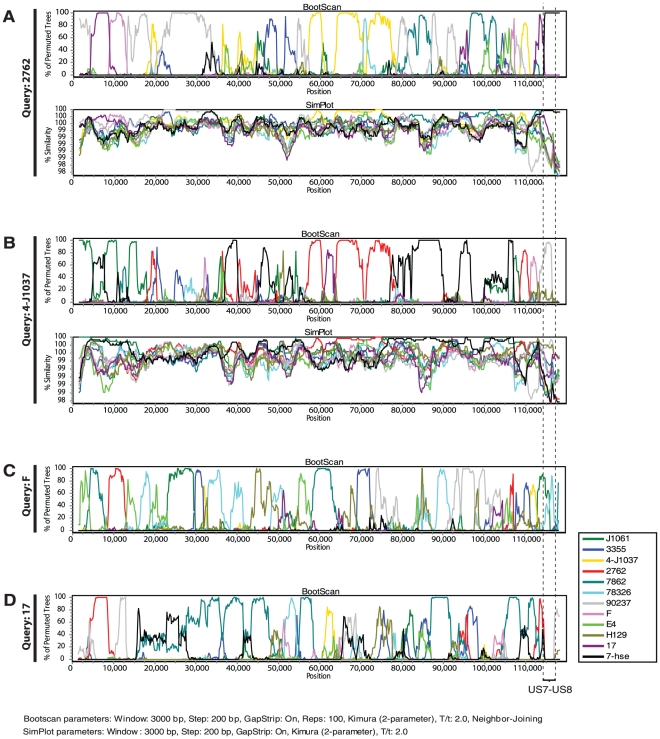
HSV-1 recombination analysis. Recombination analysis using Bootscan and SimPlot. A and B illustrate the bootscan and SimPlot analysis of query sequences 2762 and 4-J1037, respectively, which were classified as non-recombinants based on the US7–US8 regions. Bootscan plots demonstrate highly fragmented genomes as a result of recombination. Similarity plots demonstrate the sequence similarity between the query sequence and the other sequences. C and D depicts the bootscan analysis of the reference strains F and 17. Nucleotide positions refer to the sequence alignment excluding gaps and repeat regions.

To analyze and visualize recombination crossovers and the degree of fragmentation and shifting phylogenetic relationships between genomes, we used the bootscan and SimPlot methods. Results from theses analyses demonstrated highly fragmented genomes. For example, the two strains 2762 and 4-J1037, which were classified as non-recombinants when analyzing only US7 and US8, presented highly shifting phylogenetic relationships to the other strains in the genome-wide analysis (Fig. 2A and 2B). The SimPlot analysis of the same strains demonstrated a high (near 100%) sequence similarity to at least four and two putative parental strains, respectively. In addition, the bootscan analysis of the laboratory strains F and 17 revealed highly shifting phylogenetic relationships indicating massive recombination (Fig. 2C and 2D). Similar results were obtained by the bootscan analysis of the other strains (data not shown).

### Timescale of HSV-1 evolution

**Figure 3:**
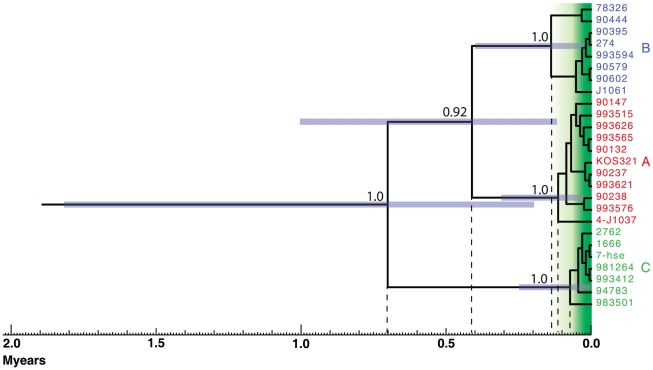
HSV-1 timescale estimation based on the genes US7 and US8. Estimated timescale for the HSV-1 evolution. Each major branch point in the phylogenetic tree is marked with a dotted line to the scale-bar below. The 95% confidence intervals for each predicted time since divergence are denoted with purple bars. The increased number of branch points in each clade is highlighted with green background. Posterior probabilities for the major clades are shown.

Due to the high frequency of recombination crossovers in the genomes, the timescale analysis of HSV-1 was restricted to the US7 to US8 genomic region, for which the three clades A, B and C are well characterized. This approach also allowed us to include additional strains that were previously presented [8]. Only strains with no recombination crossovers in this region were included in the analysis. The mean substitution rate, averaged over the whole tree in this region, was estimated to 1.82×10^−8^ nucleotide substitution per site per year. The estimated times since the most recent common ancestors were as follows: clades A, B and C: ∼710,000 years; clades A and B: ∼410,000 years; clade B: ∼137,000 years; clade A: ∼113,000 years; and clade C: ∼72,000 years (Fig. 3). Most of the branch points within each clade occurred between 70,000 and 10,000 years BP (shaded in green in Fig. 3). It should be noted that the standard deviation was rather high (purple bars in Fig. 3), indicating that the true times since divergences may differ slightly from these estimations.

### Additional phylogenetic analysis of VZV

**Figure 4:**
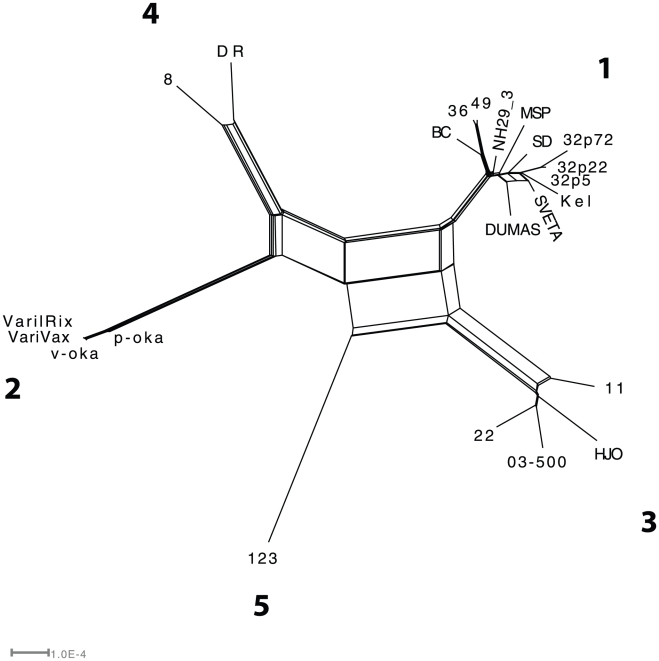
VZV phylogeny. Phylogenetic network (splits network) of available complete genome VZV sequences. The strains are divided into clades 1 to 5, and the deep lineage reticulate topology is caused by a few ancient recombination events described previously. The network also presents a reticulate pattern within clade 1 caused by a putative recent recombination event involving strain SVETA.

To extend previous analysis of VZV, we constructed a phylogenetic network based on all available complete VZV genome sequences (Fig. 4). In addition to a deep lineage reticulate pattern demonstrated previously [16], [17], [20], [21], the network constructed here, and previously by Schmidt-Chanasit and Sauerbrei [21], also demonstrates a reticulate pattern within clade 1, indicating that strain SVETA is involved in an intraclade recombination event. To analyze this further, we also applied the phi-statistics on strain SVETA and its putative parental strains Kel, Dumas, SD and MSP (the sequences of the passaged strain 32 were excluded from this analysis to minimize influences of mutations introduced during cell-passages). The result demonstrates a high statistical significance for recombination (p = 0.038) within clade 1.

## Discussion

The genome-wide analysis performed here reveals an evolutionary history of HSV-1 including an extremely high frequency of recombination events, and that all HSV-1 genomes consist of mosaic patterns of genetic segments of different evolutionary origins. The frequency of recombinants and recombination crossovers in each HSV-1 genome is hence considerably higher than previously demonstrated by the analysis of the US4 to US8 regions, supporting previous suggestions that most, or all, of the globally circulating HSV-1 strains are recombinants [8]. However, it is worth noting that the bootscan method applied here was designed to demonstrate recombination crossovers in a recombinant strain as compared to its non-recombinant parental strains. If, however, the putative parental strains are also recombinants, some of the recombination crossovers demonstrated in the query strain might instead be crossovers in any of the other strains. The frequency of recombination crossovers in each specific strain might therefore be overestimated if all strains are recombinants, which may be the case here with HSV-1. Nevertheless, the method provides an informative way to illustrate changes in phylogenetic relationships between the query strain and the other strains as a result of recombination.

In contrast to VZV, most of the detected recombination crossovers in HSV-1 are unique for each strain. It is thus likely that these recombination events are relatively recent events, except for at least one ancient recombination crossover previously demonstrated in US4 [8]. The majority of the branch points of the analyzed strains was estimated to have occurred 70,000 to 10,000 years BP (highlighted in green in Fig. 3) implying that the vast majority of the detected recombination events in HSV-1 occurred 0 to 70,000 years ago. This feature, in combination with the fact that three distinct phylogenetic clades can still be detected in short genomic stretches, suggest that strains from the three clades have been geographically separated for significant time. This hypothesis fits well with the suggested time since divergence of the different HSV-1 clades of ∼700,000 to 70,000 years BP, and the putative geographic isolation of *Homo sapiens* and other *Homo*-species during that time. Therefore, most of the HSV-1 recombination events are probably results of more recent mixtures of viral populations due to human migrations and explorer expeditions.

The high frequency of HSV-1 recombinants may appear somehow surprising since only a few ancient recombination events have been demonstrated for VZV. Complete VZV genomes can be classified into distinct phylogenetic clades, and five clades (and two additional clades that needs to be confirmed) have hitherto been demonstrated [16], [17], [19], [22]. In contrast, the extremely high frequency of HSV-1 recombinants demonstrated here emphasizes the irrelevance of assigning a complete HSV-1 genome to a specific phylogenetic clade as can be done with VZV. This is an important feature that needs to be considered in future genotyping strategies and viral genotype/phenotype association studies.

Another difference between the two viruses is that HSV-1 is at least four times more diverged than VZV. Such differences in diversity for similar viruses are commonly explained by different times since divergence and thus in this case, that HSV-1 has had significantly longer time to evolve into different clades than VZV. This assumption is based on another assumption, namely that these viruses evolve with a similar molecular clock. Since HSV-1 and VZV both are human alpha-herpesviruses with similar biology including persistency in sensory neurons, such a difference in time since divergence is paradoxical. The hypothetical shorter time scale of VZV divergence might suggest either an isolation of the virus in a subpopulation of human hosts, or a zoonotic transfer of VZV from another species. Since VZV shows a high global seroprevalence and a low mutation rate, neither of these explanations seems plausible. There might however, be another more likely explanation for the differences in divergence of HSV-1 and VZV. We suggest that these viruses may instead have started their divergence into different clades at approximately the same time-point. The formation of the different clades hence probably reflects similar co-evolution with the human host. This explanation does not necessarily exclude similar mutation rates in terms of mutations per replication cycle, but instead assumes different numbers of replication cycles (and hence also mutations) per year.

After primary infection, both viruses can reactivate from a state of latency or persistency in sensory ganglia. In a comparative study on viral presence in trigeminal ganglia from cadavers, VZV was detected in 97% and HSV-1 in 65% of the subjects [23]. While HSV-1 may reactivate frequently (up to monthly in some cases) to cause oral herpes, the reactivation of VZV in form of herpes zoster seldom occurs more than once in a lifetime. Furthermore, while HSV-1 is shed asymptomatically in saliva several times per week in almost all studied subjects, such shedding of VZV is rarely detected [24], [25]. Taken together, it is probable that HSV-1 undergoes a higher number of replication cycles per year, as compared with VZV. In addition, an early (mean 13 months) acquisition of HSV-1 as suggested by seroconversion data [26] is compatible with a spread from parents while outbreaks of chickenpox may originate from herpes zoster in the grandparental generation [27], further slowing down the evolution of VZV in comparison to HSV-1.

Despite the lower frequency of recombinants reported for VZV than for HSV-1, it is likely that the inherent ability to recombine is similar for those. As an example, during simultaneous cell culture infection with two distinct strains of another varicellovirus, the bovine herpesvirus 1, 40% of the progeny were found to be recombinants already after one round of replication [28]. These bovine herpesvirus 1 recombinants presented a highly fragmented pattern similar to the fragmentation presented here for HSV-1. Likewise, experimental infection with several VZV strains resulted in recombinants of this virus [29]. Furthermore, in the present study we demonstrate that intra-clade recombination occurs also for VZV, exemplified by strain SVETA. Thus, there may be an alternative explanation for the (in comparison to HSV-1) lower frequency of recombinant VZV strains. As suggested above, a putative lower frequency of reactivations and replication cycles per year for VZV most likely diminish the possibilities for recombination. That is, even if two VZV strains infect the same host, the probability for simultaneous replication in the same cell decreases if the number of replications and reactivations is low. Another possibility is that a primary infection of several VZV strains simultaneously is relatively uncommon as compared to HSV-1, due to the low degree of asymptomatic shedding of the former virus.

Furthermore, while all three HSV-1 clades were found to co-exist in the same geographic locations [8], [11], the VZV clades have been demonstrated to follow a specific geographic distribution [30], [31], [32], [33]. Such geographic separation of strains from different clades would be an efficient barrier to prevent intra-clade recombination.

Interestingly, recent studies indicate that this geographic separation of VZV clades is also about to fade out. An investigation of VZV in Germany demonstrates a higher genetic diversity between clinical isolates from patients with varicella than from those with zoster, an observation which the authors suggest is due to recent import of genotypes from other countries [34], [35]. Furthermore, coinfection of two VZV clades, both circulating in the same area, was recently demonstrated in a child with varicella [36]. Another evidence of geographic integration of VZV clades is that in immigrant patients with herpes zoster in East London, 30% of the strains belonged to European clades despite a history of varicella in the countries of origin of these individuals (14). This finding clearly indicates that reinfection of VZV might be common, which in turn may allow for recombination events.

Another novel possibility of VZV recombination events is the world-wide distribution of an attenuated vaccine strain [37]. This live VZV vaccine, which clusters to clade 2, is currently routinely used in for example Japan, Korea, the USA, Canada, Australia, Germany, Costa Rica, Uruguay, and Qatar, where strains from other VZV clades are predominant. This increased geographic mixing of VZV strains from different clades will undoubtedly weaken the geographic barrier for intra-clade recombination of VZV.

In conclusion we demonstrate that the evolution of HSV-1 and VZV, on a genome-wide scale, show two major differences. First, the genetic diversity of globally circulating strains is significantly higher for HSV-1 than for VZV and second; the frequency of recombinant strains is much higher for HSV-1 than for VZV. We propose that these two differences are related to biological properties regarding replication and spread of the respective infection rather than to distinct molecular clocks and different time-scales of evolution in humans. Owing to the increased mixing of previously geographically separated VZV strains, the frequency of inter-clade VZV recombinants may drastically increase in the future. Thus, the mosaic genetics of HSV-1 can be a plausible indicator on future evolutionary characteristics of VZV, provided that migration and/or vaccination alter previous geographic restriction of the spread of this virus.

## Materials and Methods

### HSV-1 strains

Clinical isolates of HSV-1 (n = 9) from USA (4 strains of which 2 were isolated from oral lesions and 2 from brains of patients with encephalitis) and from Sweden (5 strains of which one was isolated from lip and four from brains) were used to infect Vero cells at a multiplicity of infection of approximately 0.01. Cells were harvested when showing 100% cytopatic effect and lysed in 1% SDS, Tris-EDTA buffer at pH 8.0. Total DNA was purified by Proteinase K digestion and phenol/chloroform extraction and HSV-1 DNA was separated from the host DNA by NaI gradient centrifugation, as previously described [38]. Gradient-purified HSV-1 DNA was subjected to sequencing, performed via pyrosequencing on a Roche GS FLX instrument using the Titanium sequencing kits. All strains received a minimum depth of coverage of 40×. The data was assessed using the GS Assembler software as a de novo assembly as well as by mapping to the reference sequence of HSV-1 strain 17 (NC_001806) using the GS Mapper software with similar results. Each strain produced a number of high quality contigs (Q40 Plus Bases greater than 99.6%) even though the coverage greatly exceeded the 25× coverage necessary for high quality de novo assemblies. In general it was observed that the unique long and unique short regions produced single contigs whereas the repeat regions where highly fragmented and represented by numerous, relatively short contigs with multiple gaps, some spanning more than 1 kb. This did not appear to be an assembly issue per se as the mapping results produced similar results. It would appear that the sequencing libraries were underrepresented with reads in this region which may be due to a PCR bias as a result of the high G+C content of the repeat regions. The sequencing data for this study has been deposited to the short read archive in Genbank under accession number SRA035240.1.

### DNA sequence analysis

The viral DNA sequences were aligned by using ClustalW included in eBioTools and BioX 1.5, using default settings, followed by manual inspection of the complete alignment. All gaps and tandem repeat regions were excluded prior to the analysis. Genetic distances were calculated using SimPlot as a mean value in a 3000 bp window, which were slid through the genomes with a step size of 200 bp. To calculate a measure of statistical significance for recombination we used the phi-test, which has been proven to give reliable results for conserved DNA sequences [39]. The splits networks were created by using SplitsTree 4.10 [40], using the uncorrected P characters transformation (other models of characters transformation yielded only negligible differences in results). The bootscan analysis and similarity plots were performed using the SimPlot program [41] with a window size of 3000 nt and a 200 nt step size.

### Calculating time scale for HSV-1

To estimate a substitution rate for the US7 to US8 genomic region in HSV-1, and dates for the divergences into the three clades, we used the BEAST software [42]. ModelTest [43] was used to estimate the appropriate evolutionary model (Substitution model: GTR; Site heterogeneity model: Gamma; Number of gamma parameters: 4). The clock model was set to relaxed clock with uncorrected log-normal so as to account for lineage specific heterogeneity. To account for the exponential population growth, the tree prior was set to ‘Coalescent: exponential growth’. HSV-2 was included as outgroup, and the mean time since divergence of HSV-1 and HSV-2 was set to 8.45 Myears with a standard deviation of 25.000 years, which gives a central 95% range of about 4.5 to 4.4 Myears since divergence, which corresponds well to earlier suggestions of HSV-1/HSV-2 divergence [44], [45]. The MCMC was run with a chain length of 10,000,000 with sampling of parameters every 200 step. The final tree was constructed as a maximum clade credibility tree with 10% burning (the first 10% of the trees were removed prior to the final tree was built), with no posterior probability limit (all nodes annotated), and the node heights were calculated as mean heights.
